# Association of Cardiovascular Disease Mortality and Ambient Temperature Variation in Shanghai, China: Beyond Air Quality Index PM_2.5_

**DOI:** 10.3390/atmos16020119

**Published:** 2025-01-22

**Authors:** Qi Li, Shizhen Li, Ting Zhai, Shan Jin, Chunfang Wang, Bo Fang, Tian Xia

**Affiliations:** 1 Shanghai Municipal Center for Disease Control and Prevention, Department of Vital Statistics, Institute of Health Information, Shanghai 200336, China; 2 National Key Laboratory of Intelligent Tracking and Forecasting for Infectious Diseases, National Institute of Parasitic Diseases at Chinese Center for Disease Control and Prevention (Chinese Center for Tropical Diseases Research), NHC Key Laboratory of Parasite and Vector Biology, WHO Collaborating Centre for Tropical Diseases, National Center for International Research on Tropical Diseases, Shanghai 200025, China; 3 Department of Environmental Health, Harvard T.H. Chan School of Public Health, Boston, MA 02115, USA

**Keywords:** climate change, ambient temperature, cardiovascular disease, mortality

## Abstract

Evidence from megacity registry data regarding the independent association between ambient temperature and cardiovascular disease (CVD) mortality, after accounting for Particulate Matter 2.5 (PM_2.5_), remains scarce. In this study, we collected 308,116 CVD mortality cases in Shanghai from 2015 to 2020. The distributed lag non-linear model (DLNM) was utilized. The daily PM_2.5_ concentration was transformed using a natural spline (ns) function and integrated into the model for adjustment. The DLNM analysis revealed that the exposure–response curve between daily temperature and CVD mortality approximated an inverted “J” shape, consistent for both women and men. The minimum mortality temperature (MMT) for total CVD mortality was 25 °C, with an MMT of 26 °C for females and 24 °C for males. The highest relative risk (RR) of CVD mortality was 2.424 [95% confidence interval (95% CI): 2.035, 2.887] at the lowest temperature of −6.1 °C, with 2.244 (95% CI: 1.787, 2.818) for female and 2.642 (95% CI: 2.100, 3.326) for male. High temperatures exert acute and short-term effects, with the peak risk occurring on the day of exposure. In contrast, the risk from low temperature peaks on day 3 of the lag time and subsequently declines until days 16–21. This study offers evidence-based support for the prevention of temperature-induced CVD mortality.

## 1. Introduction

The Intergovernmental Panel on Climate Change (IPCC) indicates that with the massive emission of greenhouse gases, global warming, and the continuous rise of sea levels, climate change has occurred and is having an unprecedented impact worldwide [1]. According to predictions from The Lancet’s statistical data, extreme temperature events will cause approximately 250,000 deaths per year between 2030 and 2050, and by 2030, the direct cost of climate change to human health could reach USD 2 to 4 billion per year [2,3]. Climate change has increased both the average temperature and its variability [4], especially rapid temperature changes over short periods, such as extreme temperature events (cold spells and heatwaves), temperature variability, and diurnal temperature range, and the relationship between these factors and human health remains a current research hotspot [5–7].

In recent years, cardiovascular disease (CVD) has become a global public health issue that endangers human health. Data published by the World Health Organization show that in 2019, 17.9 million people worldwide died from CVD, accounting for 32% of all deaths, making it the leading cause of death globally [8]. There are many factors that affect the incidence and mortality of CVD, and environmental temperature, as an important environmental factor, is closely related to human production, life, and health [9–12]. Multiple studies have confirmed a statistically significant negative correlation between ambient temperature and blood pressure, and this correlation is stronger in patients with diseases related to CVD [13,14]. When ambient temperatures rise, peripheral blood vessels dilate, redirecting more blood flow from the viscera to the body surface to increase heat dissipation, leading to an increased cardiovascular and pulmonary load [15]. When in a cold environment, peripheral blood vessels constrict to reduce heat loss, and the heart needs to pump blood faster to maintain body temperature, resulting in increased cardiac load [16]. It is evident that the impact of ambient temperature variation and rapid temperature changes on the cardiovascular system warrants attention.

Due to differences in geography and demographic characteristics, the effects of environmental factors on CVD show strong regional specificity [17,18]. In addition, more studies have focused on the relationship between PM_2.5_ and CVD, and there is a notable absence of research addressing the influence of ambient temperature on CVD mortality [19–21]. Our study analyzed the relationship between ambient temperature and CVD mortality and the time lag of the effect by adjusting for PM_2.5_. This provides a robust framework for other researchers to follow when conducting similar studies in different regions or with different health outcomes. Given the global relevance of climate change and its impact on health, our study contributes to the broader understanding of how urban populations in different regions may be affected. The findings can inform global health initiatives and guide the development of adaptive strategies to mitigate the health impacts of climate change.

## 2. Subjects and Methods

### 2.1. Data Collection and Processing

The CVD mortality data for this study are intended to be sourced from the Death Registration Information System by Shanghai Municipal Center for Disease Control and Prevention (SCDC). This system obtains mortality information through death registration reporting, and the causes of death are reviewed and coded by professionals according to the Tenth Revision of the International Statistical Classification of Diseases and Related Health Problems (ICD-10), ensuring the accuracy of cause-of-death coding and classification. A range of quality control measures are employed to ensure the completeness and accuracy of death registration data. These measures included community physicians conducting household surveys to investigate deaths or public health physicians verifying death cases by reviewing hospital admissions, discharges, and medical records, as well as by interviewing family members. All reported information were cross-checked by SCDC staff with public security records and body disposal registration departments. The collected items for this study included sex, ethnicity, date of birth, date of death, place of residence, and underlying cause of death ICD codes.

The data meet the requirements for de-identification, with the time frame being from 1 January 2015 to 31 December 2020, and the ICD codes for the underlying cause ranging from I00 to I99. The meteorological data for this study are derived from the China Meteorological Administration’s Meteorological Information Center, including daily average temperature, daily maximum temperature, daily minimum temperature, average relative humidity, and average atmospheric pressure. To adjust for the confounding effects of air pollutants, the database will incorporate the daily average concentrations of PM_2.5_, with pollutant data sourced from the National Ecological Environment Bureau’s Air Quality Information Center.

### 2.2. Statistical Analysis

In this study, distributed lag non-linear model (DLNM) were employed to assess the mortality effects of temperature variability. The natural spline (ns) function was used for smooth fitting, and variables such as the “Day of the Week” (DOW) effect, time trend, relative humidity, and air pollutants were included in the model to control for confounding biases [22]. The model expression is as follows:

$$\left\{\begin{array}{c} Y_{t}∼\text{quasiPoisson}\left(\mu_{t},\varphi \mu_{t}\right)\\ log\left[E\left(Y_{t}\right)\right]=\alpha +\beta \cdot T_{t,\text{l}}(Z)+ns(\text{time},df=k\times n)+ns\left({PM}_{2.5t},df=k\right)\\ +ns\left({RH}_{t},df=k\right)+{DOW}_{t} \end{array}\right.$$

In the model, t represents the date. $Y_{t}$ denotes the number of deaths due to CVD. The expected value of CVD mortality is denoted by $E\left(Y_{t}\right)$, and is modeled using a quasi-Poisson distribution to account for overdispersion in the data, characterized by $u_{t}$ and $\varphi \mu_{t}$, where φ is the dispersion parameter and $\mu_{t}$ is the mean count. α is the intercept, and β is the coefficient for the temperature indicator matrix $T_{t,l}(Z)$, which employs a two-dimensional cross-basis function to model the exposure–response relationship across various lag times. ns is utilized to adjust for the non-linear effects of time trend, PM_2.5_ concentrations, and relative humidity (RH), with degrees of freedom df determined by minimizing the Akaike Information Criterion for Quasi-Poisson model (Q-AIC). Lastly, DOW_t_ is a dummy variable that captures the effect of the day of the week on the counts of circulatory system diseases. The model construction and analysis are conducted using the “dlnm” package in R software [23].

By adjusting the lag time in the model, the degrees of freedom df, covariates (such as including different air pollutant indicators), etc., the robustness of the existing model, and the goodness of fit are analyzed. If the model is sensitive to parameters, it indicates that the research results are unstable, and the findings should be interpreted with caution. We used cross-validation to select the optimal number of knots and conducted robustness checks by varying the number of knots. Residual analysis showed no systematic patterns, indicating that our model was neither overfitting nor underfitting.

Statistical analysis was conducted using software R 4.1.2 (R Foundation for Statistical Computing, Vienna, Austria) with a two-sided test level of α=0.05.

## 3. Results

### 3.1. Demographic Characteristics

Table 1 displays the distribution of daily CVD deaths and meteorological factors in Shanghai from 2015 to 2020. A total of 308, 116 CVD deaths were reported during the study period, averaging 141 deaths per day. Shanghai (31.22° N, 121.48° E) is one of China’s megacities with 25 million residents, located on the eastern coast of the Asian continent at the mouth of the Yangtze River, experiencing a subtropical monsoon climate with distinct seasons, characterized by hot and humid summers, cool to cold winters, and milder springs and autumns. The city’s climate is marked by significant temperature variations, with an average daily mean temperature of 17.58 °C and relative humidity levels averaging at 73.67%.

### 3.2. Trends in Daily Meteorological Indicators and CVD Mortality

**Figure 1.**
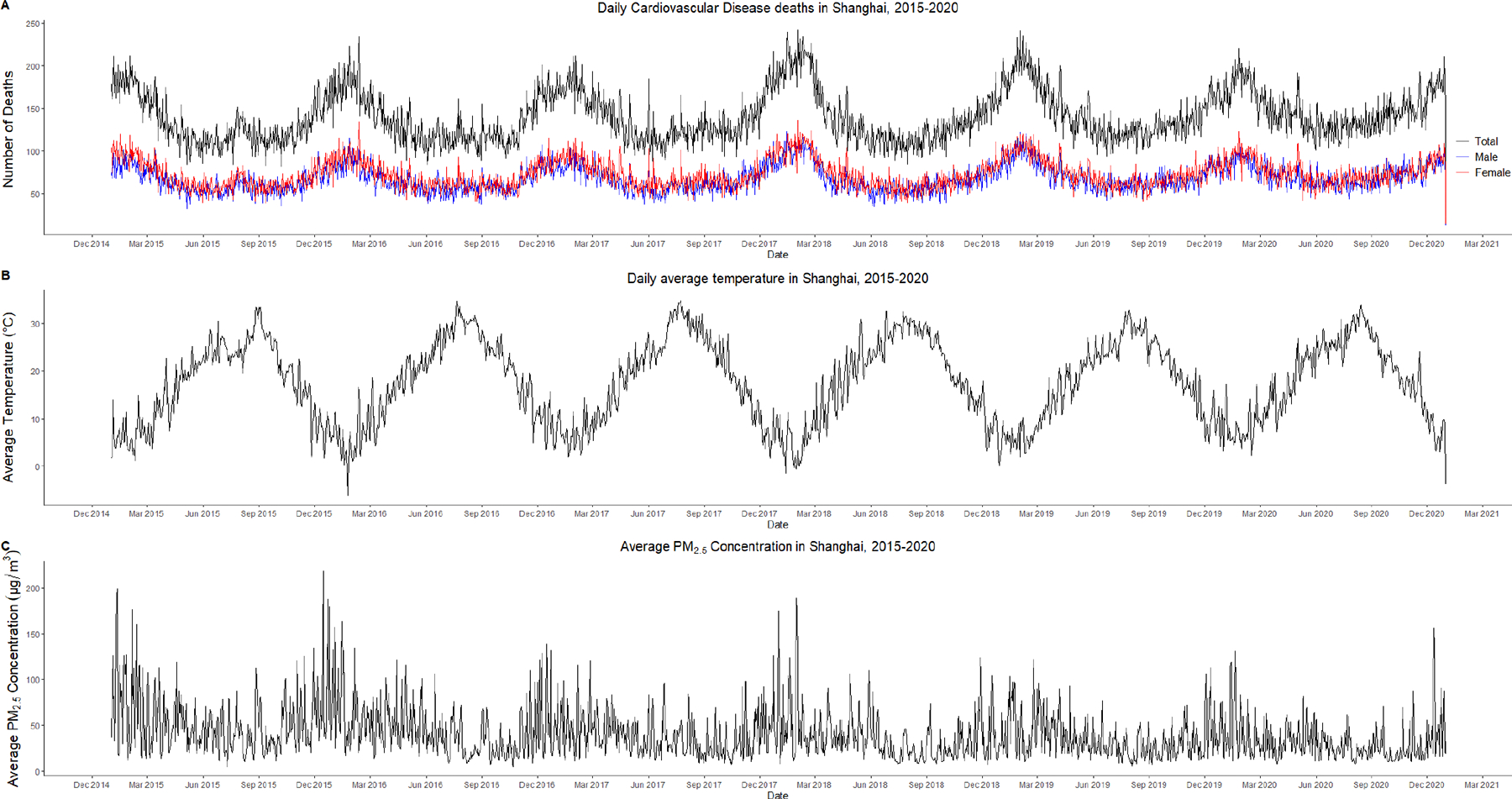
Time series plot of daily CVD mortality and changes in temperature and humidity in Shanghai from 2015 to 2020. (**A**) Daily CVD deaths in Shanghai, 2015–2020; (**B**) Daily average temperature in Shanghai, 2015–2020; (**C**) Daily average PM_2.5_ concentrations in Shanghai, 2015–2020.

During the study period, there was a clear seasonal trend in the daily CVD mortality and ambient temperature in Shanghai. From December to March each year, the daily average temperature reaches its minimum, while the daily CVD mortality reaches its peak. Interestingly, despite the variations, periods of higher PM_2.5_ concentrations occur between December and February each year (Figure 1).

### 3.3. Effects of Different Temperatures on Total CVD Mortality

**Figure 2.**
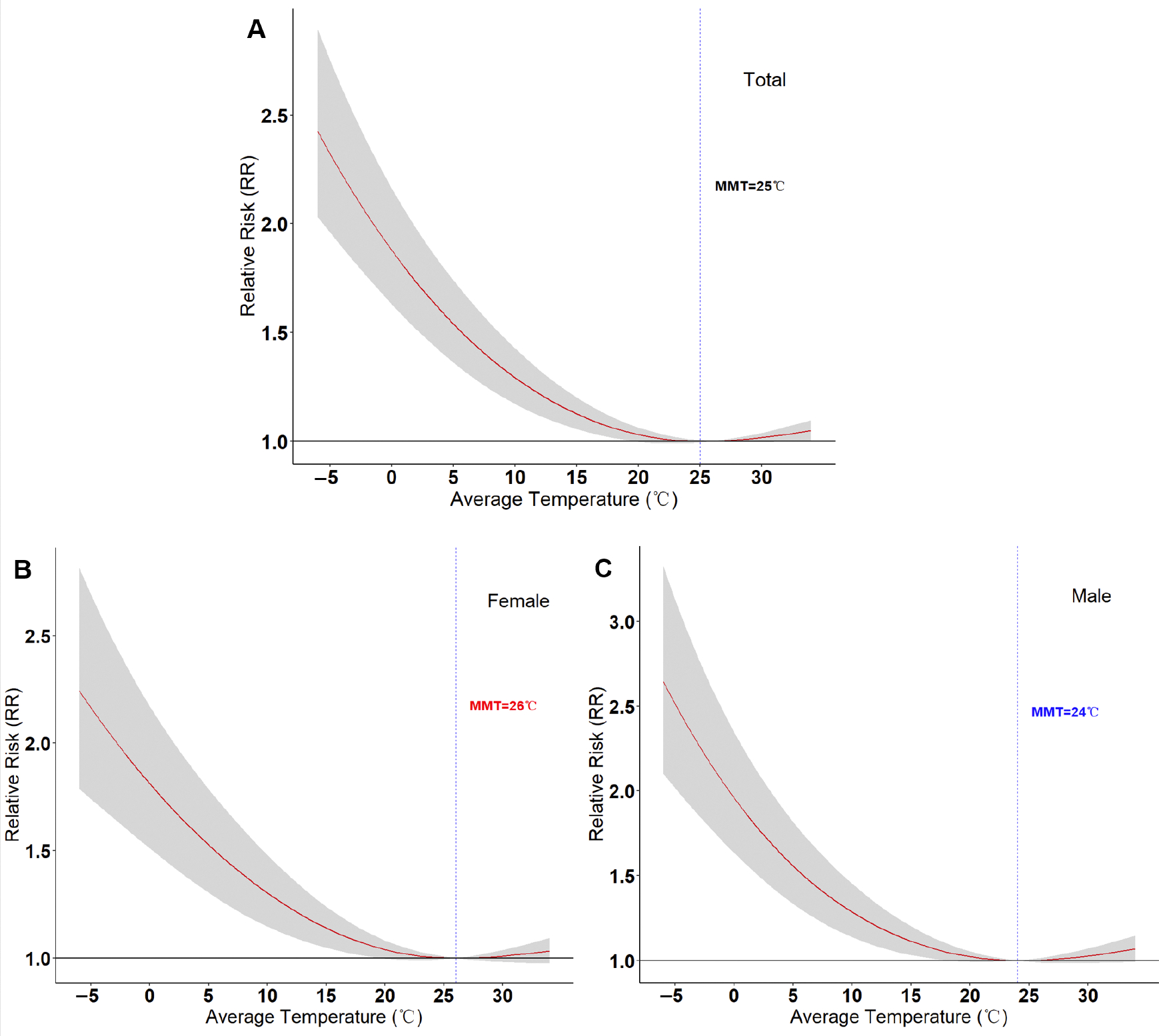
Association curve between daily average temperature and CVD deaths in Shanghai, 2015–2020. (**A**) Total CVD deaths; (**B**) CVD deaths in females; (**C**) CVD deaths in males. The grey shaded area represents the 95% confidence interval (95% CI) of the relative risk (RR) values.

Within the lag period of 0–21 days, the exposure–response curve between the daily average temperature and CVD mortality is non-linear, approximately resembling an inverted “J” shape, and this association is present in both men and women. The minimum mortality temperature (MMT) for the total number of CVD deaths is 26 °C, with an MMT of 26 °C for women and 24 °C for men. When the temperature is below the MMT, the risk of CVD death gradually increases with decreasing temperature. At the lowest temperature (−6.1 °C), the relative risk (RR) for total CVD deaths is 2.424, with a 95% confidence interval (95% CI) of (2.035, 2.887): for females it is 2.244 (1.787, 2.818), and for males it is 2.642 (2.100, 3.326). When the temperature exceeds the MMT, the risk of CVD death is not statistically significant (Figure 2).

### 3.4. Lag Effects of Ambient Temperatures on CVD Mortality

Table 2 illustrates that when the ambient temperature is below the MMT (25 °C), the risk of CVD associated with temperature exposure emerges between the second and third day post-exposure. When the temperature less than or equal to the 2.5th percentile (2.69 °C), the effect of temperature on CVD mortality gradually increases and then decreases between the 2nd and 3rd day post-exposure, persisting until the 21st day; when the temperature between the 25th percentile (10.11 °C) and the MMT (25 °C), the effect of temperature on CVD mortality gradually increases and then decreases between the 3rd and 5th day post-exposure, persisting until the 16th day. When the temperature is above the MMT (25 °C), the effect of temperature on CVD mortality is immediate on the day of exposure and rapidly diminishes within one day.

## 4. Discussion

A total of 308, 116 CVD deaths were reported in Shanghai during 2015–2020, averaging 141 deaths per day. This study discovered an inverted “J” shaped non-linear relationship between the daily average temperature and the risk of CVD mortality among Shanghai residents. Both high and low temperatures were found to increase the risk of CVD mortality. High temperatures had a short-term acute effect, while the effect of low temperatures exhibited a lag characteristic. Furthermore, extreme low temperatures had a more significant impact on CVD mortality. At the lowest temperature (−6.1 °C), the relative risk (RR) for total CVD mortality is 2.424 [95% confidence interval (95% CI) (2.035, 2.887)]: for females it is 2.244 (1.787, 2.818), and for males it is 2.642 (2.100, 3.326). This result is higher than those from Suzhou, China [OR: 1.37, 95% CI (1.30, 1.44)], and Virginia, USA [OR: 1.10, 95% CI not reported], but lower than that from Thailand [OR: 2.92, 95% CI (0.55, 5.10)]. This may be due to regional and population differences, as well as different temperature distributions [24–26].

While climate change is commonly associated with increased temperatures and global warming, there are also studies reporting that climate change is related to temperature decreases, including cold waves [27,28]. For instance, Cohen and colleagues have linked Arctic warming to an increase in cold spells in North America. Although the frequency of extreme cold days has generally declined, assessing the burden associated with high and low temperatures remains crucial, as they are closely related to the impacts of climate change [29]. Our study further confirms the importance of preventing the hazards of low temperatures to CVD patients in the context of global climate change.

This study demonstrates a non-linear, inverted “J” shaped relationship between daily average temperature and CVD mortality rates. The impact of extreme low temperatures is slower to manifest, peaking after 3–5 days and persisting for a longer duration, typically 16–21 days or more, which is consistent with other research findings [30–33]. This may be attributed to the fact that the maximum risk levels associated with low temperatures often occur a few days later and last longer, with residents tending to focus on keeping warm only on the day of the cold spell and being insufficiently attentive during the subsequent impact period. This finding also serves as a reminder to residents that protection against low temperatures should be maintained for 16 days or longer.

This study also found that the MMT for CVD is higher in females than in males, and the risk in males is higher than in females. Previous studies have shown that there are sex differences in the risk patterns of CVD mortality caused by non-optimal temperatures, with some indicating similarities between males and females [34], some showing higher rates in females [33], and others indicating higher rates in males [35]. The underlying causes of these sex differences remain unclear and should be explored in future prospective studies. Some researchers suggest that these differences may be related to factors such as thermoregulation, physiological responses, and cultural and socioeconomic factors [36,37]. For instance, influenced by China’s economic structure and culture, males generally engage in more outdoor labor in high- or low-temperature environments, while females tend to work indoors [38,39]. To some extent, this may explain the differences observed between males and females in our analysis.

### Limitations

This study has the following limitations. Firstly, since this study is based on data from Shanghai, the findings are specific. However, the urban characteristics and the environmental and health challenges are prevalent in many urban settings globally, making the findings from Shanghai relevant to other urban areas. Also, the methodology employed in this study can be adopted by other regions to analyze their local data. Secondly, since this study is based on surveillance data rather than questionnaires, the raw data do not include participants’ activity patterns (indoor and outdoor time) or behaviors, nor do they include data on occupational or environmental allergen exposure or medication use. Nevertheless, we included a sufficiently long period of data (from 2015 to 2020) to minimize the bias that these confounding factors might introduce to the results. Thirdly, due to the lack of long-term records for other environmental pollutants, carbon oxides, nitrogen oxides, hydrocarbons, etc., were not included in the analyses, and the impact of climate change requires long-term assessment using dynamic and adaptive approaches; future research should consider incorporating these factors to track changes in cardiovascular disease mortality patterns as climate conditions evolve. This will provide real-time data for public health policies.

## 5. Conclusions

This study demonstrates a significant association between ambient temperature and CVD mortality, independent of PM_2.5_, with an inverted “J” shaped exposure–response curve and an MMT of 25 °C for total CVD mortality. Notably, the MMT is higher in females than in males, yet males face a higher risk. High temperatures induce acute, short-term risks that peak on the day of exposure, while the risks associated with low temperatures peak on day 3 of lag and decline by days 16–21.

Our findings have important implications for public health policy and climate change adaptation strategies. As climate change continues to alter temperature patterns globally, our study contributes to the broader scientific literature on the health impacts of climate change. The methodology employed, including the use of DLNM and adjustment for PM_2.5_, provides a robust framework for other researchers to follow when conducting similar studies in different regions or with different health outcomes.

This study offers evidence-based support for the prevention of temperature-induced CVD mortality. It emphasizes the necessity of targeted public health interventions to reduce CVD deaths and provides valuable information for policy-making in climate change adaptation. Future research should continue to explore the complex interactions between temperature, air quality, and cardiovascular health, as well as the potential modifying effects of socio-economic factors and individual behaviors. Long-term monitoring and analysis of these relationships will be crucial for developing effective strategies to protect public health in the face of a changing climate.

## Figures and Tables

**Table 1.** Distribution of daily cardiovascular disease (CVD)deaths and meteorological factors in Shanghai, 2015–2020.

Number of Daily Death	Mean	SD	Min	P_1_	P_2.5_	P_25_	P_50_	P_75_	P_97.5_	P_99_	Max

Male	67.7	15.47	13	40.91	44	56	65	77	101	108	123
Female	72.86	16.67	14	44	46.775	61	70	84	109	116.09	136

Meteorological Factors											

Daily Average Temperature (°C)	17.58	8.56	−6.1	1.2	2.69	10.11	18.5	24.53	31.62	32.9	34.8
Daily Average Relative Humidity (%)	73.67	12.36	28.63	42.98	46.97	65.34	74.31	83	94	95.76	100
Daily Average PM_2.5_ Concentrations (μg/m^3^)	39.76	27.14	5	9	10	20	32	51	112	132.09	218

**Table 2.** The effects of different temperature distributions on CVD mortality with different lag days in Shanghai, 2015–2020.

Number of Lag Days	Temperature and Distribution (°C)					

	Min	P2.5	Mean	MMT	P97.5	Max

	−6.1	2.69	17.58	25	31.62	34.8

Lag0	0.830 (0.769, 0.896)	0.816 (0.781, 0.853)	0.916 (0.897, 0.935)	Ref.	1.069 (1.035, 1.104)	1.102 (1.048, 1.159)
Lag1	1.048 (0.987, 1.112)	1.004 (0.971, 1.040)	0.981 (0.966, 0.997)	Ref.	1.029 (1.003, 1.056)	1.045 (1.003, 1.088)
Lag2	1.121 (1.085, 1.157)	1.065 (1.045, 1.085)	1.005 (0.997, 1.014)	Ref.	1.005 (0.991, 1.019)	1.010 (0.988, 1.032)
Lag3	1.126 (1.094, 1.158)	1.074 (1.057, 1.092)	1.013 (1.005, 1.021)	Ref.	0.994 (0.982, 1.007)	0.993 (0.974, 1.013)
Lag4	1.111 (1.082, 1.141)	1.072 (1.056, 1.089)	1.016 (1.009, 1.024)	Ref.	0.991 (0.980, 1.003)	0.988 (0.970, 1.006)
Lag5	1.090 (1.072, 1.110)	1.065 (1.054, 1.076)	1.017 (1.012, 1.021)	Ref.	0.991 (0.984, 0.998)	0.988 (0.977, 0.999)
Lag6	1.073 (1.058, 1.089)	1.058 (1.048, 1.067)	1.016 (1.012, 1.020)	Ref.	0.992 (0.986, 0.997)	0.989 (0.980, 0.998)
Lag7	1.061 (1.044, 1.077)	1.051 (1.041, 1.061)	1.015 (1.011, 1.019)	Ref.	0.992 (0.986, 0.998)	0.989 (0.979, 0.999)
Lag8	1.052 (1.034, 1.069)	1.045 (1.034, 1.055)	1.014 (1.009, 1.018)	Ref.	0.992 (0.986, 0.999)	0.989 (0.979, 1.000)
Lag9	1.045 (1.029, 1.061)	1.039 (1.029, 1.049)	1.012 (1.008, 1.017)	Ref.	0.992 (0.986, 0.999)	0.990 (0.980, 0.999)
Lag10	1.041 (1.028, 1.055)	1.034 (1.025, 1.043)	1.011 (1.007, 1.014)	Ref.	0.992 (0.987, 0.998)	0.990 (0.981, 0.998)
Lag11	1.038 (1.027, 1.050)	1.030 (1.022, 1.037)	1.009 (1.006, 1.012)	Ref.	0.993 (0.989, 0.997)	0.990 (0.983, 0.996)
Lag12	1.036 (1.026, 1.047)	1.026 (1.019, 1.033)	1.008 (1.005, 1.010)	Ref.	0.993 (0.989, 0.996)	0.990 (0.984, 0.995)
Lag13	1.034 (1.023, 1.044)	1.022 (1.015, 1.029)	1.006 (1.004, 1.009)	Ref.	0.993 (0.990, 0.997)	0.990 (0.985, 0.996)
Lag14	1.032 (1.021, 1.043)	1.019 (1.012, 1.026)	1.005 (1.002, 1.008)	Ref.	0.994 (0.990, 0.997)	0.991 (0.985, 0.997)
Lag15	1.029 (1.018, 1.041)	1.016 (1.009, 1.024)	1.004 (1.001, 1.007)	Ref.	0.994 (0.990, 0.998)	0.992 (0.985, 0.998)
Lag16	1.027 (1.015, 1.039)	1.014 (1.007, 1.022)	1.003 (1.000, 1.006)	Ref.	0.995 (0.991, 0.999)	0.993 (0.986, 0.999)
Lag17	1.025 (1.013, 1.036)	1.012 (1.005, 1.020)	1.002 (0.999, 1.006)	Ref.	0.996 (0.991, 1.000)	0.994 (0.987, 1.000)
Lag18	1.022 (1.011, 1.034)	1.011 (1.003, 1.018)	1.002 (0.999, 1.005)	Ref.	0.996 (0.992, 1.001)	0.995 (0.989, 1.001)
Lag19	1.020 (1.009, 1.031)	1.009 (1.002, 1.017)	1.001 (0.998, 1.004)	Ref.	0.997 (0.994, 1.001)	0.996 (0.990, 1.002)
Lag20	1.017 (1.007, 1.027)	1.008 (1.002, 1.015)	1.001 (0.998, 1.004)	Ref.	0.998 (0.995, 1.002)	0.998 (0.993, 1.003)
Lag21	1.015 (1.006, 1.024)	1.008 (1.001, 1.014)	1.000 (0.998, 1.003)	Ref.	0.999 (0.997, 1.002)	0.999 (0.995, 1.004)

## Data Availability

The datasets used or analyzed during the current study are available from the corresponding author on reasonable request.

## Abbreviations

The following abbreviations are used in this manuscript:

CVD: Cardiovascular disease
PM_2.5_: Particulate Matter 2.5
DLNM: Distributed lag non-linear model
ns: Natural spline
MMT: Minimum mortality temperature
RR: Relative risk
95% CI: 95% confidence interval
IPCC: Intergovernmental Panel on Climate Change
ICD-10: The 10th Revision of the International Statistical Classification of Diseases and Related Health Problems
SCDC: Shanghai Municipal Center for Disease Control and Prevention
RH: Relative humidity
Q-AIC: Akaike Information Criterion for Quasi-Poisson model
DOW: Day of the week

## References

[1] Watts N; Amann M; Arnell N; Ayeb-Karlsson S; Belesova K; Boykoff M; Byass P; Cai W; Campbell-Lendrum D; Capstick S; The 2019 report of The Lancet Countdown on health and climate change: Ensuring that the health of a child born today is not defined by a changing climate. Lancet 2019, 394, 1836–1878. 31733928 10.1016/S0140-6736(19)32596-6 PMC7616843

[2] Goshua A; Gomez J; Erny B; Burke M; Luby S; Sokolow S; LaBeaud AD; Auerbach P; Gisondi MA; Nadeau K Addressing Climate Change and Its Effects on Human Health: A Call to Action for Medical Schools. Acad. Med. J. Assoc. Am. Med. Coll. 2021, 96, 324–328. 10.1097/ACM.0000000000003861 33239537

[3] Romanello M; McGushin A; Di Napoli C; Drummond P; Hughes N; Jamart L; Kennard H; Lampard P; Solano Rodriguez B; Arnell N; The 2021 report of the Lancet Countdown on health and climate change: Code red for a healthy future. Lancet 2021, 398, 1619–1662. 34687662 10.1016/S0140-6736(21)01787-6 PMC7616807

[4] Zandalinas SI; Fritschi FB; Mittler R Global Warming, Climate Change, and Environmental Pollution: Recipe for a Multifactorial Stress Combination Disaster. Trends Plant Sci. 2021, 26, 588–599. 33745784 10.1016/j.tplants.2021.02.011

[5] Lee JY; Kim H; Gasparrini A; Armstrong B; Bell ML; Sera F; Lavigne E; Abrutzky R; Tong S; Coelho M; Predicted temperature-increase-induced global health burden and its regional variability. Environ. Int. 2019, 131, 105027. 31351381 10.1016/j.envint.2019.105027

[6] Yang Y; Li L; Chan PW; Zhou Q; Sheng B Intercomparison of Local Warming Trends of Shanghai and Hong Kong Based on 120-Year Temperature Observational Data. Int. J. Environ. Res. Public Health 2022, 19, 6494. 35682078 10.3390/ijerph19116494 PMC9180144

[7] Wang H; Geng M; Schikowski T; Areal A; Hu K; Li W; Coelho M; Saldiva P; Sun W; Zhou C; Increased Risk of Influenza Infection During Cold Spells in China: National Time Series Study. JMIR Public Health Surveill. 2024, 10, e55822. 39140274 10.2196/55822 PMC11336504

[8] WHO. Cardiovascular Diseases (CVDs). Available online: https://www.who.int/news-room/fact-sheets/detail/cardiovascular-diseases-(cvds) (accessed on 15 January 2025).

[9] Dimitrova A; Ingole V; Basagaña X; Ranzani O; Milà C; Ballester J; Tonne C Association between ambient temperature and heat waves with mortality in South Asia: Systematic review and meta-analysis. Environ. Int. 2021, 146, 106170. 33395923 10.1016/j.envint.2020.106170

[10] Gasparrini A; Guo Y; Hashizume M; Lavigne E; Zanobetti A; Schwartz J; Tobias A; Tong S; Rocklöv J; Forsberg B; Mortality risk attributable to high and low ambient temperature: A multicountry observational study. Lancet 2015, 386, 369–375. 26003380 10.1016/S0140-6736(14)62114-0 PMC4521077

[11] Martínez-Solanas È; Quijal-Zamorano M; Achebak H; Petrova D; Robine JM; Herrmann FR; Rodó X; Ballester J Projections of temperature-attributable mortality in Europe: A time series analysis of 147 contiguous regions in 16 countries. Lancet Planet. Health 2021, 5, e446–e454. 34245715 10.1016/S2542-5196(21)00150-9

[12] Rahman MM; Garcia E; Lim CC; Ghazipura M; Alam N; Palinkas LA; McConnell R; Thurston G Temperature variability associations with cardiovascular and respiratory emergency department visits in Dhaka, Bangladesh. Environ. Int. 2022, 164, 107267. 35533532 10.1016/j.envint.2022.107267 PMC11213361

[13] Fan P; Xue X; Hu J; Qiao Q; Yin T; Yang X; Chen X; Hou Y; Chen R Ambient temperature and ambulatory blood pressure: An hourly-level, longitudinal panel study. Sci. Total Environ. 2023, 864, 160854. 36521627 10.1016/j.scitotenv.2022.160854

[14] Modesti PA The shifted focus of interest in the temperature-blood pressure relationship: From load to variability. Hypertens. Res. 2021, 44, 1548–1550. 34480135 10.1038/s41440-021-00729-8

[15] Johnson JM; Minson CT; Kellogg DL Jr. Cutaneous vasodilator and vasoconstrictor mechanisms in temperature regulation. Compr. Physiol. 2014, 4, 33–89. 24692134 10.1002/cphy.c130015

[16] Bhatnagar A Environmental Determinants of Cardiovascular Disease. Circ. Res. 2017, 121, 162–180. 28684622 10.1161/CIRCRESAHA.117.306458 PMC5777598

[17] Yim G; Wang Y; Howe CG; Romano ME Exposure to Metal Mixtures in Association with Cardiovascular Risk Factors and Outcomes: A Scoping Review. Toxics 2022, 10, 116. 35324741 10.3390/toxics10030116 PMC8955637

[18] Kazi D; Katznelson E; Liu C-L; Al-Roub N; Chaudhary R; Young D; McNichol M; Mickley L; Kramer D; Cascio W; Climate Change and Cardiovascular Health: A Systematic Review. JAMA Cardiol. 2024, 9, 748. 38865135 10.1001/jamacardio.2024.1321 PMC11366109

[19] Hayes RB; Lim C; Zhang Y; Cromar K; Shao Y; Reynolds HR; Silverman DT; Jones RR; Park Y; Jerrett M; PM_2.5_ air pollution and cause-specific cardiovascular disease mortality. Int. J. Epidemiol. 2020, 49, 25–35. 31289812 10.1093/ije/dyz114 PMC7124502

[20] Peralta AA; Castro E; Danesh Yazdi M; Kosheleva A; Wei Y; Schwartz J Low-level PM_2.5_ exposure, Cardiovascular and Non-accidental Mortality, and Related Health Disparities in 12 U.S. States. Epidemiology 2024. Epub ahead of print. 10.1097/EDE.0000000000001820 PMC11785480 39575927

[21] Alexeeff SE; Deosaransingh K; Van Den Eeden S; Schwartz J; Liao NS; Sidney S Association of Long-term Exposure to Particulate Air Pollution With Cardiovascular Events in California. JAMA Netw. Open 2023, 6, e230561. 36826819 10.1001/jamanetworkopen.2023.0561 PMC9958530

[22] Gasparrini A; Armstrong B; Kenward MG Distributed lag non-linear models. Stat. Med. 2010, 29, 2224–2234. 20812303 10.1002/sim.3940 PMC2998707

[23] Gasparrini A Distributed Lag Linear and Non-Linear Models in R: The Package dlnm. J. Stat. Softw. 2011, 43, 1–20. PMC3191524 22003319

[24] Hua Y; Zhou L; Liu F; Yang H; Wang L; Huang C; Liu C; Lu Y; Wang H; Kan H Association between ambient temperature and cause-specific mortality: An individual-level case-crossover study in Suzhou, China. Ecotoxicol. Environ. Saf. 2024, 282, 116687. 38981395 10.1016/j.ecoenv.2024.116687

[25] Davis RE; Roney PC; Pane MM; Johnson MC; Leigh HV; Basener W; Curran AL; DeMarcy B; Jang J; Schroeder C; Climate and human mortality in Virginia, 2005–2020. Sci. Total Environ. 2023, 894, 164825. 37343846 10.1016/j.scitotenv.2023.164825

[26] Denpetkul T; Phosri A Daily ambient temperature and mortality in Thailand: Estimated effects, attributable risks, and effect modifications by greenness. Sci. Total Environ. 2021, 791, 148373. 34126499 10.1016/j.scitotenv.2021.148373

[27] Garcia RA; Cabeza M; Rahbek C; Araújo MB Multiple dimensions of climate change and their implications for biodiversity. Science 2014, 344, 1247579. 24786084 10.1126/science.1247579

[28] Zhao Q; Guo Y; Ye T; Gasparrini A; Tong S; Overcenco A; Urban A; Schneider A; Entezari A; Vicedo-Cabrera AM; Global, regional, and national burden of mortality associated with non-optimal ambient temperatures from 2000 to 2019: A three-stage modelling study. Lancet Planet. Health 2021, 5, e415–e425. 34245712 10.1016/S2542-5196(21)00081-4

[29] Cohen J; Agel L; Barlow M; Garfinkel CI; White I Linking Arctic variability and change with extreme winter weather in the United States. Science 2021, 373, 1116–1121. 34516838 10.1126/science.abi9167

[30] Alahmad B; Khraishah H; Royé D; Vicedo-Cabrera AM; Guo Y; Papatheodorou SI; Achilleos S; Acquaotta F; Armstrong B; Bell ML; Associations Between Extreme Temperatures and Cardiovascular Cause-Specific Mortality: Results From 27 Countries. Circulation 2023, 147, 35–46. 36503273 10.1161/CIRCULATIONAHA.122.061832 PMC9794133

[31] Ma Y; Zhou L; Chen K Burden of cause-specific mortality attributable to heat and cold: A multicity time-series study in Jiangsu Province, China. Environ. Int. 2020, 144, 105994. 32745780 10.1016/j.envint.2020.105994

[32] Wan K; Feng Z; Hajat S; Doherty RM Temperature-related mortality and associated vulnerabilities: Evidence from Scotland using extended time-series datasets. Environ. Health 2022, 21, 99. 36284320 10.1186/s12940-022-00912-5 PMC9594922

[33] Achebak H; Rey G; Lloyd S; Quijal-Zamorano M; Méndez-Turrubiates R; Ballester J Ambient temperature and risk of cardiovascular and respiratory adverse health outcomes: A nationwide cross-sectional study from Spain. Eur. J. Prev. Cardiol. 2024, 31, 1080–1089. 38364198 10.1093/eurjpc/zwae021

[34] Yang M; He Q; Wu K; Wu Q; Bai Z; Cheng Q; Hu J; Wang H; Su H; Xing X; Urban-rural disparities in the short-term effects of cold and heat on myocardial infarction mortality in Anhui Province, China. Am. J. Epidemiol. 2024. Epub ahead of print. 10.1093/aje/kwae108 38872336

[35] Ban J; Xu D; He MZ; Sun Q; Chen C; Wang W; Zhu P; Li T The effect of high temperature on cause-specific mortality: A multi-county analysis in China. Environ. Int. 2017, 106, 19–26. 28554097 10.1016/j.envint.2017.05.019 PMC5760246

[36] Alahmad B; Shakarchi AF; Khraishah H; Alseaidan M; Gasana J; Al-Hemoud A; Koutrakis P; Fox MA Extreme temperatures and mortality in Kuwait: Who is vulnerable? Sci. Total Environ. 2020, 732, 139289. 32438154 10.1016/j.scitotenv.2020.139289

[37] Atkins W; McKenna Z; Jarrard C; Foster J; Crandall C Thermoregulatory Responses to Very Hot and Dry Heat Exposure: Interactions Between Age and Sex. Physiology 2024, 39, 726.

[38] Xia Y; Liu Z; Hu B; Rangarajan S; Ah Tse L; Li Y; Wang J; Hu L; Wang Y; Xiang Q; Associations of outdoor fine particulate air pollution and cardiovascular disease: Results from the Prospective Urban and Rural Epidemiology Study in China (PURE-China). Environ. Int. 2023, 174, 107829. 36934571 10.1016/j.envint.2023.107829

[39] Xia X; Chan KH; Niu Y; Liu C; Guo Y; Ho KF; Yim SHL; Wang B; Doherty A; Avery D; Modelling personal temperature exposure using household and outdoor temperature and questionnaire data: Implications for epidemiological studies. Environ. Int. 2024, 192, 109060. 39401479 10.1016/j.envint.2024.109060 PMC7616742

